# Identification of the Novel Tumor Suppressor Role of FOCAD/miR-491-5p to Inhibit Cancer Stemness, Drug Resistance and Metastasis via Regulating RABIF/MMP Signaling in Triple Negative Breast Cancer

**DOI:** 10.3390/cells10102524

**Published:** 2021-09-24

**Authors:** Wei-Chieh Huang, Hsiang-Cheng Chi, Shiao-Lin Tung, Po-Ming Chen, Ya-Chi Shih, Yi-Ching Huang, Pei-Yi Chu

**Affiliations:** 1 Graduate Institute of Integrated Medicine, China Medical University, NO91, Hsueh-Shih Road, Taichung 40402, Taiwan; jeff20628@gmail.com (W.-C.H.); nonbalance@gmail.com (H.-C.C.); yaoming9@yahoo.com.tw (P.-M.C.); ali5800717@gmail.com (Y.-C.S.); ychuang5176@gmail.com (Y.-C.H.); 2 Chinese Medicine Research Center, China Medical University, NO91, Hsueh-Shih Road, Taichung 40402, Taiwan; 3 Department of Hematology and Oncology, Ton-Yen General Hospital, Hsinchu County 30210, Taiwan; sonoratung@gmail.com; 4 Department of Nursing, Hsin Sheng Junior College of Medical Care and Management, Taoyuan 33858, Taiwan; 5 Graduate Institute of Biomedical Engineering, National Chung Hsing University, Taichung 40402, Taiwan; 6 School of Medicine, College of Medicine, Fu Jen Catholic University, New Taipei 242, Taiwan; 7 Department of Pathology, Show Chwan Memorial Hospital, Changhua 500, Taiwan; 8 Department of Health Food, Chung Chou University of Science and Technology, Changhua 510, Taiwan; 9 National Institute of Cancer Research, National Health Research Institutes, Tainan 704, Taiwan

**Keywords:** TNBC, miR-491-5p, FOCAD, RABIF, MMP

## Abstract

Triple negative breast cancer (TNBC) possesses poor prognosis mainly due to development of chemoresistance and lack of effective endocrine or targeted therapies. MiR-491-5p has been found to play a tumor suppressor role in many cancers including breast cancer. However, the precise role of miR-491-5p in TNBC has never been elucidated. In this study, we reported the novel tumor suppressor function of FOCAD/miR-491-5p in TNBC. High expression of miR-491-5p was found to be associated with better overall survival in breast cancer patients. We found that miR-491-5p could be an intronic microRNA processed form *FOCAD* gene. We are the first to demonstrate that both miR-491-5p and FOCAD function as tumor suppressors to inhibit cancer stemness, epithelial-mesenchymal transition, drug resistance, cell migration/invasion, and pulmonary metastasis etc. in TNBC. MiR-491-5p was first reported to directly target Rab interacting factor (RABIF) to downregulate RABIF-mediated TNBC cancer stemness, drug resistance, cell invasion, and pulmonary metastasis via matrix metalloproteinase (MMP) signaling. High expression of RABIF was found to be correlated with poor clinical outcomes of breast cancer and TNBC patients. Our data indicated that miR-491-5p and RABIF are potential prognostic biomarkers and targeting the novel FOCAD/miR-491-5p/RABIF/MMP signaling pathway could serve as a promising strategy in TNBC treatment.

## 1. Introduction

Among female patients, breast cancer remains the most commonly occurring cancer and causes the second highest number of cancer deaths [1]. As much as 24% of all newly diagnosed breast cancers are triple-negative breast cancer (TNBC). TNBC, the most aggressive type of breast cancer, is characterized by the absence of estrogen receptors (ER) and progesterone receptors (PR) and the amplification of human epidermal growth factor receptor 2 (HER2) [2,3,4]. However, chemoresistance to initial chemotherapy, frequent recurrence, and lack of effective endocrine or targeted therapies lead to a poor prognosis for TNBC [3,5,6]. While a few targeted therapies, including inhibitors of the mammalian target of rapamycin (mTOR), epidermal growth factor receptor (EGFR), and vascular endothelial growth factor (VEGF), have been recently applied in TNBC treatment, the effects remain unsatisfactory. There is, therefore, an urgent need to explore new therapeutic targets [6].

MicroRNAs (miRNAs) are a class of small non-coding RNAs containing about 20–25 nucleotides in length, which can affect biological process via repression of translation or reducing the stability of mRNA [7]. Aberrant expression of miRNAs has been found in many cancers to play the role of either tumor suppressors or oncogenes [3,8]. MiRNA-491-5p (miR-491-5p) which is located at the site of chromosome 9p21.3 has been reported to serve as a tumor suppressor in various cancers including breast cancer, cervical cancer, ovarian cancer, gastric cancer, pancreatic cancer, bladder cancer, oral squamous cell carcinoma (OSCC), and osteosarcoma [9,10,11,12,13,14,15,16,17,18,19,20]. Although the tumor suppressor role of miR-491-5p in breast cancer has been depicted in several studies such as preventing proliferation, apoptosis, migration and invasion by targeting ZNF-703 [10,12] and inhibiting cell growth by targeting JMJD2B [9], the precise role of miR-491-5p in TNBC has never been elucidated.

Rab (Ras-related in brain) interacting factor (RABIF), also named MSS4 (mammalian suppressor of Sec4), has been isolated as a mammalian counterpart of yeast DSS4 (dominant suppressor of Sec4) to act as a guanine nucleotide exchange factor (GEF) for guanosine triphosphate hydrolases (RabGTPase) [21,22,23]. Human RABIF gene was found to be located at chromosome 1q32.1 and high level of RABIF mRNA expression was once reported in pancreatic cancer tissues [24,25]. RABIF has been indicated to exhibit chaperone activity for GLUT4 exocytosis [26]. RABIF was also found to interact with RAB13 to promote cell migration via directing RAB13 GTPase activity [27]. High expression of RABIF protein was identified to protect cells from stress-induced apoptosis through binding to eukaryotic translation initiation factor 3 subunit f (elF3f) protein [28]. The interaction of RABIF with α-integrin subunits was shown to be important for regulating MMP-2 and MMP-9 activation and remodeling of fibronectin [29]. Nevertheless, the actual character of RABIF in regulating cancer development and metastasis has remained obscure. 

This study identifies a novel tumor suppressor role for FOCAD/miR-491-5p in TNBC. We are the first to show that overexpression of miR-491-5p has an inhibitory effect on cancer stemness, the epithelial-mesenchymal transition (EMT), drug resistance, anchorage-independent growth, cell invasion, and pulmonary metastasis in TNBC. We also found high expression of miR-491-5p correlated with better overall survival in breast cancer patients. Further, we demonstrated that miR-491-5p could be an intronic miRNA processed from the *FOCAD (KIAA1797)* gene [30] and identified that the focadhesin (FOCAD) protein played a tumor suppressor role in repressing cancer stemness, cell migration, tissue invasion, and pulmonary metastasis in TNBC. We revealed that miR-491-5p also directly targeted RABIF and downregulated RABIF-mediated TNBC cancer stemness, drug resistance, cell invasion, and pulmonary metastasis via matrix metalloproteinase (MMP)-2 and MMP-9 signaling. High expression of RABIF was associated with poor clinical outcomes in TNBC patients. Our data elucidated that miR-491-5p and RABIF are potential prognostic biomarkers of TNBC, and targeting the novel FOCAD/miR-491-5p/RABIF/MMP signaling pathway could be a promising approach for TNBC treatment.

## 2. Material and Methods 

### 2.1. Cell Culture and Sphere Forming Assay

We used Dulbecco’s modified eagle medium (DMEM; Invitrogen, Carlsbad, CA, USA) to grow human TNBC and breast epithelial cell lines, including MDA-MB-231, Hs578T and MCF10A cells, supplemented with 10% fetal bovine serum (FBS; Invitrogen, Carlsbad, CA, USA). The cells were incubated at 37 °C in 5% CO_2_. To obtain spheres, TNBC cells were cultured in a stem cell selective condition plating cells in Corning Costar ultra-low attachment 6-well plates (Sigma-Aldrich Inc., St Louis, MO, USA) at a density of 1 × 10^4^ cells per well with 1 mL of serum-free PSGro hESC/iPSC growth medium (System Biosciences, Palo Alto, CA, USA).

### 2.2. Transwell Migration and Invasion Assay

In brief, we seeded 5 × 10^4^ cells into a 6-μm Transwell insert (BD Biosciences) sitting on a 24-well plate. Next, we added 0.5 mL DMEM with 10% FBS. Following a 24-h incubation, the cells that had migrated were stained with 0.5% crystal violet staining solution for 20 min. The invasion assay was the same protocol after replacing the 6-μm Transwell insert with a Matrigel-coated Transwell insert (BD Biosciences). We used ImageJ software to quantify the migrated/invaded cells (NIH, Bethesda, MD, USA). A detailed description of the procedures has been provided in a previous study [19].

### 2.3. Western Blotting Analysis

Cells were lysed in RIPA buffer (1% TritonX-100, 50 mM pH:7.4 Tris-HCl, 150 mM NaCl_2_, and 0.1% SDS) prior to SDS-PAGE electrophoresis. The proteins were transferred from the gel to a methanol-activated PVDF membrane at 55 V for three hours. The PVDF membrane was washed twice with 1X TBST and 5% non-fat milk was used to block the membrane for one hour at room temperature. Next, specific primary antibodies were applied to the membrane during the hybridization step, and their positions were identified using HRP-conjugated secondary antibodies.

### 2.4. Short Interfering RNA (siRNA), Expression Vector of RABIF and Their Transfection

Twenty-four hours prior to the transfection, one million TNBC cells were seeded in six-well dishes. We obtained non-specific and human RABIF, FOCAD and MMP-9 smart-pool targeting siRNAs from Dharmacon, Inc. The indicated siRNAs, control siRNAs, pre-miR-491-5p or pre-anti-miR-491-5p, and control miRNAs were introduced into TNBC cells using Lipofectamine RNAiMAX (Invitrogen, Carlsbad, CA, USA). After 48 h, the transfected cells were harvested for a western blotting analysis.

The full-length RABIF, FOCAD cDNA, and FOCAD-exon4/5 (which included the sequences of exon 4, intron 4, and exon 5) were cloned into the pCDNA3.1-control plasmid. Both the MDA-MB-231 and Hs578T cells were transfected with pCDNA3.1-puro-RABIF, and puromycin (Sigma-Aldrich) was employed to select stable RABIF-expressing cancer cell lines. Lipofectamine 2000 (Invitrogen) and Lipofectamine RNAiMAX (Invitrogen) were used to transfect the cells.

### 2.5. Soft Agar Assay

A six-well plate was loaded with 1 mL of preheated DMEM with 10% FBS containing 0.5% agarose. To create the base agar, the plate was placed in an incubator at 37 °C for the medium to harden. For the upper layer, 5 × 10^4^ cells were quickly mixed with 1 mL of the preheated DMEM with 10% FBS containing 0.25% agarose (not exceeding 40 °C) and loaded above the bottom layer. The plate was immediately placed back in the incubator and kept at 37 °C for two weeks. Colonies were stained with 0.05% (*w*/*v*) iodonitrotetrazolium chloride (Sigma) for two days before the endpoint. ImageJ software (NIH, Bethesda, MD, USA) was used to quantify the number of colonies.

### 2.6. MTS Cell Proliferation Assay

Twenty-four hours prior to the MTS assay, cells (with a density of about 3 × 10^3^ per well) were seeded in a 96-well plate. We used the CellTiter96^®^ MTS assay kit (Promega, CA, USA) to analyze cell viability and followed the manufacturer instructions. The experiments were performed in triplicates and repeated thrice.

### 2.7. In Vitro Cytotoxicity Assay

Twenty-four hours prior to the drug treatment, cells (with a density of about 6 × 10^3^ per well) were seeded in the 96-well plate. Docetaxel or carboplatin were used to treat the cells using the indicated dosage for 48 h. We determined cell viability using the CellTiter96^®^ cell proliferation assay kit (Promega, Wisconsin, CA, USA) and followed all manufacturer instructions. GraphPad Prism software was used to calculate the IC_50_ value of cells treated with and without docetaxel or carboplatin. All experiments were performed in triplicates and repeated thrice.

### 2.8. ELISA Analysis

We measured the levels of secreted MMP2/9 by seeding mock-, RABIF- and FOCAD-siRNA-treated cells and the indicated plasmids into six-well dishes at a density of 5 × 10^5^ cells. Following a 48-h incubation, the medium was collected for an ELISA analysis, and cells were harvested for a Bradford assay to normalize samples. An MMP2/9 ELISA analysis was performed using the MMP2/9 kit (Catalog:KHC3081/BMS2016-2, ThermoFisher) and following manufacturer protocols. An absorbance microplate reader (an ELx800 spectrophotometer, BioTek, Bunker Hills, Medina, OH, USA) was used to determine optical density at 450 nm.

### 2.9. In Vivo Metastasis Assays

The tail veins of mice (six to eight weeks old) were intravenously injected with 1 × 10^6^ MDA-MB-231 cells. The mice were monitored for 30–60 days before sacrifice. Thereafter, the lung tissue was removed, fixed (formaldehyde solution 4%, MERCK), paraffin-embedded, serially sectioned, weighed, and stained using hematoxylin and eosin (H&E). All animal experiments were acquired through protocols approved by the Institutional Review Board approval (Approval date: 5 July 2019).

### 2.10. Web Server Survival Analysis

Kaplan-Meier plots were generated through the automatic selection of the optimal cutoff values between lower and upper quartiles, which formed the high and low expression groups (https://kmplot.com/analysis/, 1 July 2021).

### 2.11. Statistical Analysis

We conducted Student’s *t*-tests to compare the control and experimental groups. The relapse-free survival and overall survival results are presented as Kaplan–Meier survival curves. All data are presented as mean ± s.e. (standard error). Differences with *p* < 0.05 (* *p* < 0.05, ** *p* < 0.01 and *** *p* < 0.001) were considered statistically significant.

## 3. Results

### 3.1. MiR-491-5p Correlates with Longer Overall Survival and Suppresses Cancer Stemness, Epithelial-Mesenchymal Transition (EMT) and Drug Resistance in TNBC

**Figure 1:**
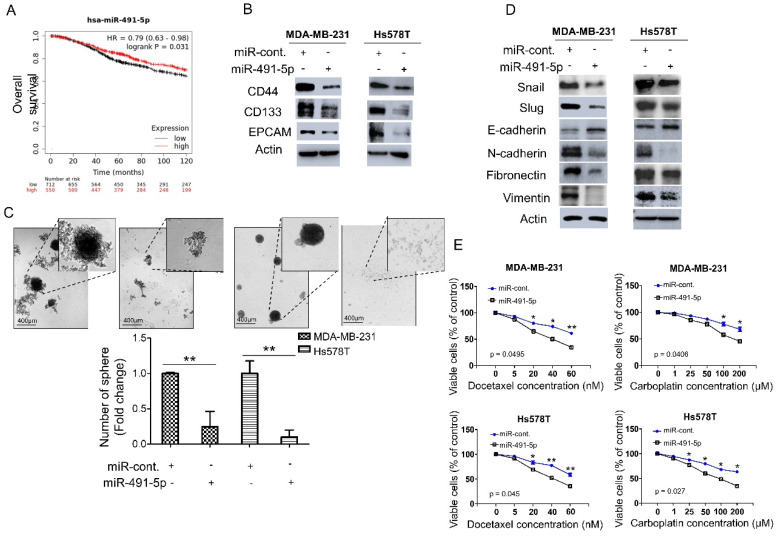
miR-491-5p correlates with better overall survival and inhibits cancer stemness, EMT and drug resistance in TNBC. (**A**) Analysis of Kaplan–Meier plotter database for miR-491-5p expression in association with the overall survival of 1262 breast cancer patients. (**B**) Western blot showing protein expression levels of breast cancer stem cell markers in MDA-MB-231 and Hs578T cells transfected with miR-491-5p or control. Actin served as the internal control. (**C**) Observations of sphere formation under stem cell selective conditions after 8 days of culturing MDA-MB-231 and Hs578T cells transfected with miR-491-5p or control. Representative images are shown. Histograms represent means ± s.e. from three independent experiments (* *p* < 0.05, ** *p* < 0.01). (**D**) Western blot analysis of protein expression levels of EMT markers in MDA-MB-231 and Hs578T cells transfected with miR-491-5p or control. Actin served as the internal control. (**E**) MTS assay showing the dose-dependent growth inhibition of MDA-MB-231 and Hs578T cells transfected with miR-491-5p or control upon continuous docetaxel or carboplatin exposure at indicated concentrations for 48 h. Each dosage point represents means ± s.e. from three independent experiments (* *p* < 0.05, ** *p* < 0.01). All experiments were performed in triplicates and done at least thrice.

We analyzed the expression levels of miR-491-5p in breast cancer patients to explore the correlation between miR-491-5p expression and clinical outcomes. We applied the Kaplan–Meier plotter (https://kmplot.com/analysis/, 1 July 2021) to online databases and selected the best cut-off points for high and low expression groups for a Kaplan–Meier survival analysis [31]. Compared with breast cancer patients with lower miR-491-5p expression (*p* = 0.031), those with higher miR-491-5p expressions were significantly associated with longer overall survival (Figure 1A). We further examined the functional role of miR-491-5p in regulating cancer stemness in vitro since TNBC reportedly demonstrates highly chemoresistant behavior and cancer stem cells (CSCs) play important roles in cancer initiation, metastasis, recurrence, and chemoresistance [6,32,33]. We conducted a western blotting analysis to study two TNBC lines, MDA-MB-231 and Hs578T, transfected with miR-491-5p to identify important breast CSC makers such as CD44, CD133, and EPCAM (epithelial cell adhesion molecule) [34,35]. Overexpression of miR-491-5p in MDA-MB-231 and Hs578T cells resulted in decreased protein expression of CD44, CD133, and EPCAM compared with the control cells (Figure 1B). We tested whether miR-491-5p impacts TNBC’s sphere-forming ability by culturing MDA-MB-231 and Hs578T cells transfected with control constructs or miR-491-5p under stem cell selective conditions (described in the Section 2). Floating spheres that had confluent, rounded, and three-dimensional configurations often expanded to a diameter of 50–100 μM after being cultured for five to eight days (Figure 1C, top). We visually counted the number of spheres under the microscope after culturing for eight days and found a decrease in the number of spheres when miR-491-5p was overexpressed (Figure 1C, bottom).

Recent reports have highlighted the relationship between CSCs and the EMT and the role it plays in tumor progression and therapeutic resistance [36,37]. Thus, this study explored the influence of miR-491-5p expression on EMT markers. The analysis revealed that compared with the control cells, the MDA-MB-231 and Hs578T cells transfected with miR-491-5p showed increased expression of E-cadherin (an epithelial marker) and the decreased expression of mesenchymal markers, including Snail, Slug, N-cadherin, fibronectin, and vimentin (Figure 1D) [38]. Taxanes and platinum compounds that target DNA repair complexes are standard chemotherapeutic agents in TNBC treatment [39]. Our MTS assay showed that overexpression of miR-491-5p increased chemosensitivity towards docetaxel and carboplatin in both MDA-MB-231 and Hs578T cells (Figure 1E). In sum, the findings indicate a novel tumor suppressor role for miR-491-5p in regulating cancer stemness, the EMT, and drug resistance in TNBC.

### 3.2. MiR-491-5p Significantly Inhibits TNBC Anchorage-Independent Growth, Cell Invasion and Pulmonary Metastasis in TNBC

**Figure 2:**
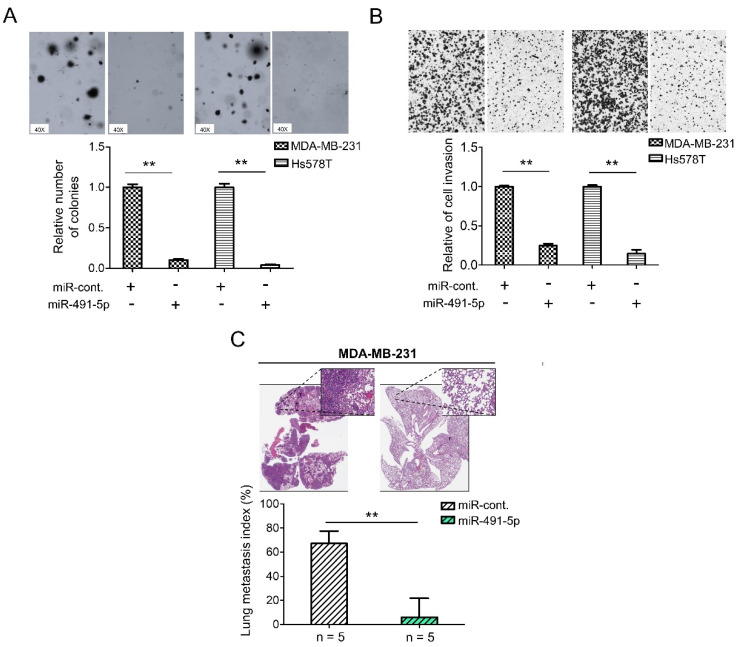
miR-491-5p suppresses 3D colony formation, cell invasion, and pulmonary metastasis in TNBC. (**A**) Overexpression of miR-491-5p reduced the colony-forming abilities of TNBC cells in a 3D soft agar culture (40×, bright field) (top). Representative images are shown. Quantitative results from the 3D soft agar assay (bottom). (**B**) Analysis of the effect of miR-491-5p on invasion ability of TNBC cells using Boyden chamber assay. The representative photographs of the invaded cells from different treatments are shown as pictures and the quantitative data are shown by histograms. (**C**) Lung metastasis of five CB17-SCID mice in each group was significantly inhibited after tail vein injection of MDA-MB-231 cells transfected with miR-491-5p shown by histologic staining and lung metastasis index. All histograms represent means ± s.e. from three independent experiments (** *p* < 0.01).

To further explore the functional roles of miR-491-5p in TNBC, we have implemented the following in vitro and in vivo experiments. To evaluate the relative expression levels of miR-491-5p in TNBC, the non-tumorigenic breast epithelial cell line, MCF10A, was used for comparison. The mRNA expression levels of miR-491-5p decreased in both MDA-MB-231 and Hs578T cells when compared with MCF10A cells by qRT-PCR analysis (Figure S1). We then applied a 3D Matrigel culture to best recapitulate tumor growth in vivo to investigate if miR-491-5p affects TNBC cell growth within a three-dimensional (3D) setting. The analysis revealed that miR-491-5p markedly inhibited anchorage-independent growth in both MDA-MB-231 and Hs578T cells (Figure 2A). Distant metastasis is a challenge in breast cancer and is particularly prevalent in TNBC. A comparison of TNBC and non-TNBC patients indicated that the former were susceptible to an increased risk of early metastasis, resulting in lower five-year survival [40,41]. Thus, we further examined if miR-491-5p affects TNBC’s invasion and metastatic abilities in vitro and in vivo. A Boyden chamber assay showed that the overexpression of miR-491-5p significantly suppressed the invasion ability of both the MDA-MB-231 and Hs578T cells (Figure 2B). Next, we employed an experimental metastasis model via a tail vein injection of 1 × 10^6^ MDA-MB-231 cells transfected with control or miR-491-5p cells into five C.B-17 severe-combined immunodeficient (CB17-SCID) female mice in each group to examine if miR-491-5p regulated TNBC metastasis in vivo. Using histologic staining and a lung metastasis index, we found that the overexpression of miR-491-5p remarkably decreased pulmonary metastasis compared with the control group (Figure 2C). The data presented thus far reinforce the tumor suppressor role of miR-491-5p in inhibiting anchorage-independent growth, cell invasion, and pulmonary metastasis in TNBC.

### 3.3. MiR-491-5p Could Be an Intronic miRNA Processed from FOCAD Gene and FOCAD Regulates Cancer Stemness, Migration/Invasion and Lung Metastasis in TNBC

**Figure 3:**
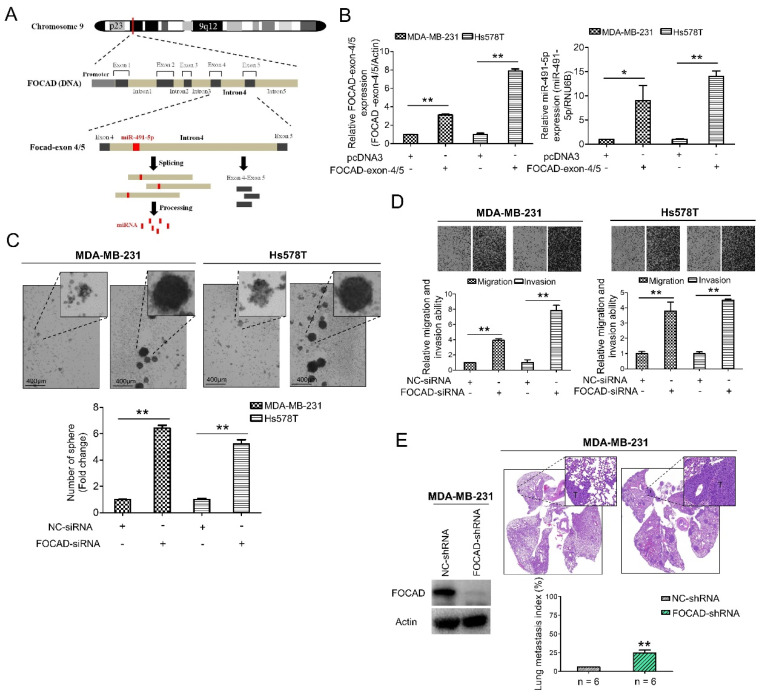
Identification of FOCAD and its functional roles in TNBC. (**A**) MiR-491-5p coding pre-sequence is located in intron 4 of *FOCAD* gene. FOCAD-exon 4/5 plasmid was generated through cloning nucleotide sequences containing exon 4, intron 4 and exon 5. (**B**) Left, the mRNA expression levels of FOCAD-exon 4/5 plasmid which was transfected into both MDA-MB-231 and Hs578T cells measured using qRT-PCR analysis. Right, the effect of overexpression of FOCAD-exon 4/5 plasmid on endogenous miR-491-5p mRNA expression in MDA-MB-231 and Hs578T cells measured using qRT-PCR analysis. (**C**) Observations of spheres forming under stem cell selective conditions after 8 days of culturing MDA-MB-231 or Hs578T cells transfected with FOCAD-siRNA or control. Representative images are shown, and quantitative data are presented as histograms (**D**) Boyden chamber assay of FOCAD-siRNA’s effect on the migration and invasion abilities of TNBC cells. Representative photographs of migrated or invaded cells from different treatments are shown, and the quantitative data are provided as histograms. (**E**) Left, Western blotting analysis of FOCAD protein expression in MDA-MB-231 cells transfected with the FOCAD-shRNA or NC-shRNA. Actin was served as an internal control. Right, lung metastasis of six CB17-SCID mice in each group was significantly augmented after tail vein injection of MDA-MB-231 cells transfected with FOCAD-shRNA shown by histologic staining and lung metastasis index. All histograms represent means ± s.e. from three independent experiments (* *p* < 0.05, ** *p* < 0.01).

The host gene of miR-491-5p, *FOCAD (KIAA1797)* gene, was recognized as a tumor suppressor gene in glioma cells [42]. *FOCAD* gene was found to be located at chromosome 9p21.3 containing the whole nucleotide sequence of miR-491-5p in its intron 4 area via comparison using Basic Local Alignment Search Tool (BLAST) (https://blast.ncbi.nlm.nih.gov/Blast.cgi, 1 July 2021) (Figure 3A). FOCAD-exon 4/5 plasmid can be generated through cloning nucleotide sequences plasmid containing exon 4, intron 4 and exon 5, and miR-491-5p can be produced from splicing and processing of FOCAD exon 4/5 plasmid (Figure 3A). This finding indicated that miR-491-5p could be an intronic miRNA processed form intron 4 area of *FOCAD* gene rather than being transcribed from a separate mRNA. Interestingly, both miR-491-5p and FOCAD has been reported to regulate FOCAD adhesion signaling in OSCC and glioma cells, respectively [19,30]. On the basis of these findings, we further explore the possible tumor suppressor role of FOCAD/miR-491-5p in TNBC.

A quantitative real-time polymerase chain reaction (qRT-PCR) analysis determined that the transfection of the FOCAD-exon 4/5 plasmid into both the MDA-MB-231 and Hs578T cells increased the mRNA expression of miR-491-5p in these two lines (Figure 3B). More specifically, we tested three FOCAD-siRNAs to determine their inhibitory efficacy through an analysis of the FOCAD mRNA and protein levels in MDA-MB-231 cells (Figure S2). The highest knockdown effect in inhibiting FOCAD mRNA and protein expression levels was reported for FOCAD-siRNA-3, which was used in the subsequent experiments (Figure S2). Interestingly, although transfection of FOCAD-exon 4/5 plasmid increased mRNA expression of miR-491-5p (Figure 3B), no significant decrease of mRNA expression level of miR-491-5p was found by inhibition of FOCAD (Figure S3).

A study was conducted to obtain insight on the functional role of FOCAD. FOCAD knockdown significantly increased the sphere-forming ability in both MDA-MB-231 and Hs578T cells (Figure 3C). Transfection with miR-491-5p markedly suppressed the formation of spheres, while cotransfection with FOCAD-siRNA and miR-491-5p partially nullified the suppression effect of miR-491-5p in Hs578T cells (Figure S4A). Further, the suppression of FOCAD in MDA-MB-231 and Hs578T cells enhanced cell migration and invasion ability (Figure 3D). Overexpression of miR-491-5p resulted in decreased cell invasion ability in Hs578T cells, cotransfection with FOCAD-siRNA and miR-491-5p partially rescued the abolishment effect of miR-491-5p (Figure S4B).

Next, we examined the influence of FOCAD on TNBC metastasis in vivo. We employed an experimental metastasis model, and injected 1 × 10 [6] MDA-MB-231 cells transfected with control or specific FOCAD small hairpin RNA (FOCAD-shRNA) via the tail vein of six CB17-SCID female mice. Compared with the control group, the shRNA-based knockdown of FOCAD increased pulmonary metastasis, as indicated by histologic staining and the lung metastasis index (Figure 3E). In summary, miR-491-5p was found to be an intronic miRNA of the *FOCAD* gene. In addition, FOCAD played a tumor suppressor role in regulating cancer stemness, migration/invasion, and lung metastasis in TNBC. The extent of the differences in regulatory abilities between FOCAD and miR-491-5p were also observed.

### 3.4. RABIF Is a Direct Target of MiR-491-5p

**Figure 4:**
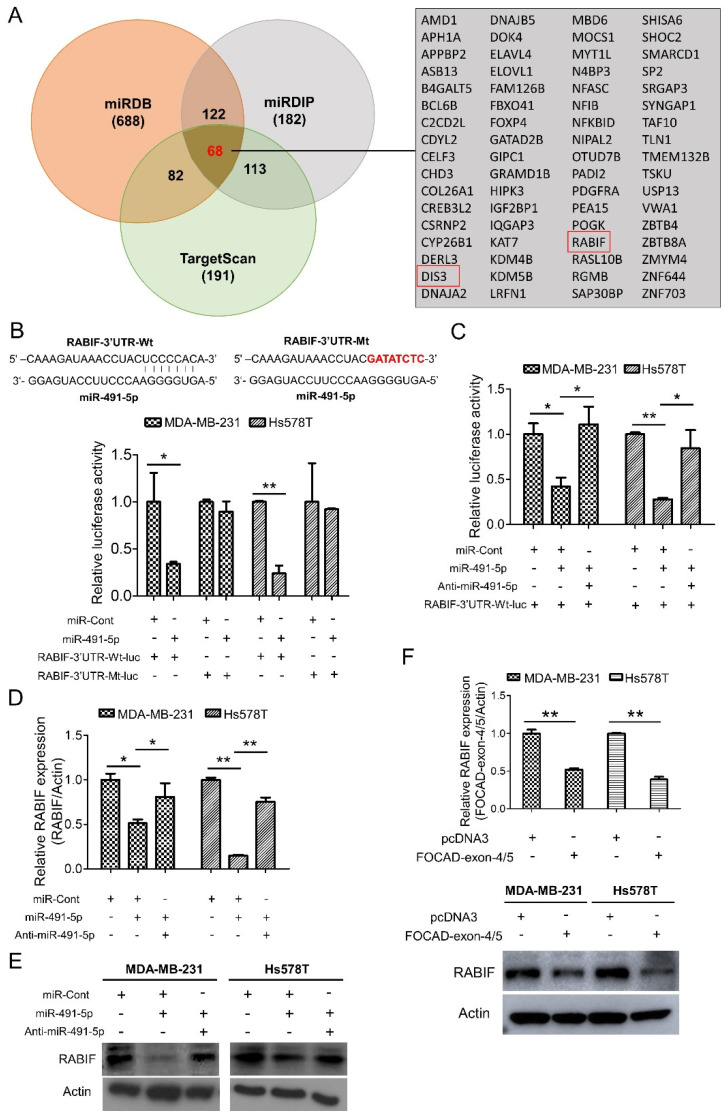
Identification of RABIF as the direct target of miR-491-5p. (**A**) Left, the number of potential targets of miR-491-5p determined by three bioinformatics algorithms (miRDB, miRDIP, and TargetScanHuman 7.2) is shown by Venn diagram. Right, the detailed list of 68 potential targets of miR-491-5p. (**B**) Effect of miR-491-5p on luciferase activity of RABIF-3′UTR-Wt-luc (wild type) and RABIF-3′UTR-Mt-luc (mutant) in MDA-MB-231 and Hs578T cells. Top, the RABIF-3′UTR-Wt-luc sequence and the RABIF-3′UTR-Mt-luc sequence in which the sequence in red was mutagenized to abolish the binding between miR-491-5p and RABIF-3′UTR. Bottom, luciferase reporter assay showed decreased activity of more than 50% after cotransfection with miR-491-5p and RABIF-3′UTR-Wt-luc in MDA-MB-231 and Hs578T cells. The activity of RABIF-3′UTR-Mt-luc was not significantly affected by miR-491-5p. (**C**) The expressions of luciferase activity of RABIF-3′UTR-Wt-luc (wild type) in MDA-MB-231 and Hs578T cells upon transfection with the indicated plasmids. (**D**) The mRNA expression levels of RABIF were measured by qRT–PCR from MDA-MB-231 and Hs578T cells transfected with the indicated plasmids. (**E**) The protein expression levels of RABIF as reflected by Western blotting analysis in MDA-MB-231 and Hs578T cells transfected with the indicated plasmids are shown. Actin was served as an internal control. (**F**) The mRNA (top) and the protein (bottom) expression levels of RABIF were measured by qRT–PCR and Western blotting analysis from MDA-MB-231 and Hs578T cells transfected with the indicated plasmids. All histograms represent means ± s.e. from three independent experiments (* *p* < 0.05, ** *p* < 0.01). All experiments were performed in triplicates and were done at least three times.

To elucidate the molecular mechanism of miR-491-5p to affect TNBC tumor growth, three online bioinformatics algorithms including TargetScanHuman 7.2, MicroRNA Target Prediction Database (miRDB) and microRNA Data Integration Portal (miRDIP) were used for searching the more accurate potential targets of miR-491-5p [43,44,45]. A total of 68 potential targets of miR-491-5p were determined in the intersection area of the three bioinformatics algorithms as shown in the Venn diagram with a detailed list (Figure 4A). RABIF and DIS3 were particularly identified from the above 68 potential targets via analyzing using the “guanyl-nucleotide exchange factor activity” functional pathway of Gene Ontology (GO) program (Figure 4A). Since RABIF acts as a GEF and GEF has been previously reported to be involved in cancer cell migration, invasion, and metastasis [24,46,47,48], we then choose RABIF as the most potential target of miR-491-5p for further investigation.

We examined if RABIF is a direct target of miR-491-5p by constructing a wild-type luciferase-RABIF-3′UTR (RABIF-3′UTR-Wt-luc) plasmid and a mutant plasmid (RABIF-3′UTR-Mt-luc), where the putative miR-491-5p binding site was mutated (Figure 4B, top). The luciferase reporter assay revealed that cotransfectingMDA-MB-231 and Hs578T cells with anti-miR-491-5p can reverse the decrease in RABIF-3′UTR-Wt-lucluciferase activity caused by miR-491-5p (Figure 4C). The assay also showed that compared with controls, miR-491-5p generated a greater than 50% suppression in the luciferase activity of the RABIF-3′UTR-Wt-luc, whereas miR-491-5p had little suppression effect on the luciferase activity of the RABIF-3′UTR-Mt-luc reporter in either the MDA-MB-231 or Hs578T cells (Figure 4B, bottom). In addition, the transfection of miR-491-5p significantly reduced RABIF, and cotransfection with anti-miR-491-5p reduced the suppressive effects at both the transcriptional and translational levels in either the MDA-MB-231 or Hs578T cells, as indicated by qRT–PCR and western blotting analyses (Figure 4D,E). The confirmation of the functional activity of anti-miR-491-5p on sphere-forming and cell invasion ability was demonstrated in Figure S5.

Similarly, transfection with FOCAD-exon 4/5 plasmid resulted in the repression of RABIF expression at the mRNA and protein levels in the MDA-MB-231 and Hs578T cells (Figure 4F). Our findings indicate that miR-491-5p directly targets RABIF and downregulates its mRNA and protein expression.

### 3.5. RABIF Correlates with Poor Clinical Outcomes and Promotes TNBC Cancer Stemness, Drug Resistance, Cell Invasion and Pulmonary Metastasis, Which Are Inhibited by MiR-491-5p

**Figure 5:**
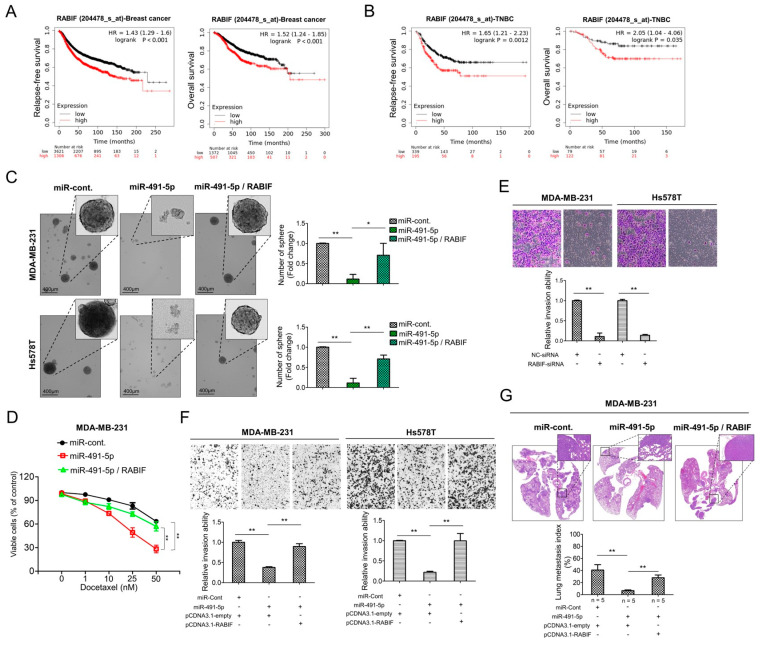
RABIF correlates with poor clinical outcomes and its oncogenic functions can be reverted by miR-491-5p in TNBC. (**A**) Analysis of Kaplan Meier plotter database for RABIF expression in association with relapse-free survival in 4929 breast cancer patients (left) and overall survival in 1879 breast cancer patients (right). (**B**) Analysis of Kaplan Meier plotter database for RABIF expression in association with relapse-free survival in 534 TNBC patients (left) and overall survival in 201 TNBC patients (right). (**C**) Observations of sphere formation under stem cell selective conditions after 8 days of culturing MDA-MB-231 or Hs578T cells transfected with the indicated plasmids (left). Representative images are shown. Quantification of spheres after 8 days of culturing (right). (**D**) MTS assay showing the dose-dependent growth inhibition of MDA-MB-231 cells transfected with the indicated plasmids upon continuous docetaxel exposure at indicated concentrations for 48 h. Each dosage point represents means ± s.e. from three independent experiments (* *p* < 0.05, ** *p* < 0.01). (**E**) Boyden chamber assay showing the effect of RABIF-siRNA on TNBC cells’ invasion ability. Representative photographs of invaded cells from different treatments are shown, and quantitative data are denoted by histograms. (**F**) Boyden chamber assay of the invasion ability of TNBC cells transfected with the indicated plasmids or vectors. Representative photographs show invasive cells under different treatment conditions. Quantitative data are denoted by histograms. (**G**) Lung metastasis of five CB17-SCID mice in each group was monitored after tail vein injection of stably RABIF-overexpressing MDA-MB-231 cells or the control cells which were both transfected with miR-491-5p or the control shown by histologic staining and lung metastasis index. All histograms represent means ± s.e. from three independent experiments (* *p* < 0.05, ** *p* < 0.01).

We examined the correlation between the RABIF expression levels and clinical outcomes. We determined the survival rates of breast cancer and TNBC patients according to RABIF expression levels by applying the Kaplan–Meier plotter. The results revealed that shorter relapse-free survival (*p* < 0.001) and overall survival (*p* < 0.001) were significantly associated with breast cancer patients with higher RABIF expression than those with lower RABIF expression (Figure 5A). Similarly, increased expression of RABIF also correlated with shorter durations of relapse-free survival (*p* = 0.0012) and overall survival (*p* = 0.035) in TNBC patients (Figure 5B). Given the lack of clarity regarding the role of RABIF in breast cancer, particularly TNBC, we further explored the functional roles of RABIF in TNBC. We first examined if RABIF promoted the sphere-forming ability of MDA-MB-231 and Hs578T cells and if miR-491-5p could suppress this ability. Following an eight-day culture of the MDA-MB-231 and Hs578T cells in stem cell selective conditions, we observed that transfection with miR-491-5p significantly decreased sphere formation, whereas cotransfection with RABIF could partially nullify the suppression effect of miR-491-5p (Figure 5C). Next, we investigated whether drug resistance was impacted by RABIF and miR-491-5p. We found that following transfection with miR-491-5p, the MDA-MB-231 cells became more chemosensitive to continuous docetaxel exposure in varying doses, whereas the cotransfection of RABIF increased chemoresistance, as shown by the MTS assay (Figure 5D). To assess cell invasion ability, we tested three RABIF-siRNAs for their inhibitory efficacy by analyzing the RABIF protein levels in MDA-MB-231 cells (Figure S6). The highest knockdown effect, inhibiting RABIF protein expression levels, was reported for RABIF-siRNA-3, which was used in the subsequent experiments (Figure S6). Further, a Boyden chamber assay highlighted that RABIF’s knockdown effect significantly reduced the invasion ability of the MDA-MB-231 and Hs578T cells (Figure 5E). Next, we prepared pCDNA3.1-RABIF or pCDNA3.1-empty vector transfected stable MDA-MB-231 and Hs578T cell lines to examine if RABIF and miR-491-5p enhanced or inhibited the invasion and metastatic abilities in TNBC in vivo and in vivo. We found that transfection with miR-491-5p decreased the protein expression level of RABIF in the MDA-MB-231 and Hs578T cells transfected with pCDNA3.1-empty vector (control cells). However, a western blotting analysis showed that miR-491-5p failed to downregulate the protein expression level of RABIF in the stably RABIF-overexpressing MDA-MB-231 and Hs578T cells transfected with the pCDNA3.1-RABIF vector (Figure S7). When compared with the control cells, we found that the inhibition of invasion ability by the transfection of miR-491-5p was impeded in stably RABIF-overexpressing MDA-MB-231 and Hs578T cells (Figure 5F). In an experimental metastasis model, we implanted 1 × 10^6^ stably RABIF-overexpressing MDA-MB-231 cells or control cells, both of which were transfected with miR-491-5p or the control, via a tail vein injection into five CB17-SCID female mice of each group. Histological staining and the lung metastasis index showed that the suppression of pulmonary metastatic ability by transfection of miR-491-5p was hindered in stably RABIF-overexpressing MDA-MB-231 cells when compared with the control cells (Figure 5G). Thus, our data demonstrate that miR-491-5p targets RABIF by inhibiting its promotion of sphere formation, drug resistance, cell invasion, and pulmonary metastasis. Moreover, RABIF is a marker of poor prognosis in TNBC.

### 3.6. Matrix Metalloproteinase (MMP)-2 and MMP-9 Are Downstream Signaling Molecules of FOCAD/MiR-491-5p/RABIF Axis

Degradation of extracellular matrix (ECM) proteins is an important process in cancer cell invasion and metastasis and the MMP enzyme family plays pivotal roles in cleavage and degradation of the ECM molecules [29,49,50]. Among the MMP family members, MMP-2 and MMP-9 are gelantinase to be responsible for degradation of collagens, elastin, aggrecan and fibronectin [51]. Previous reports indicated that MMP-2 and MMP-9 were related to tumor invasion and metastasis [50,51]. As previously mentioned, RABIF was found to bind α-integrin to regulate activation of MMP-2 and MMP-9 in ECM remodeling process [29], hence we decided to investigate if MMP-2 and MMP-9 are downstream signaling molecules of FOCAD/miR-491-5p/RABIF axis.

**Figure 6:**
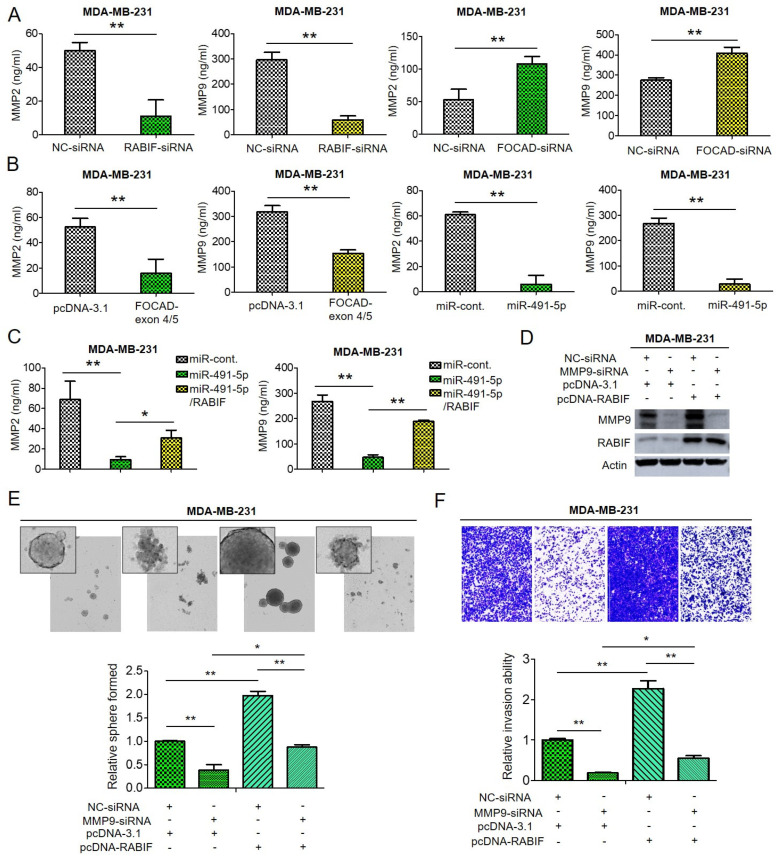
The effect of FOCAD/miR-491-5p/RABIF axis on the expression of MMP-2 and MMP-9. (**A**) Quantification of MMP-2 and MMP-9 levels in MDA-MB-231 cells transfected with RABIF-siRNA (left) or FOCAD-siRNA (right) measured using ELISA analysis. (**B**) The quantities of MMP-2 and MMP-9 in MDA-MB-231 cells transfected with FOCAD-exon 4/5 plasmid (left) or miR-491-5p (right) were measured by ELISA analysis. (**C**) The quantities of MMP-2 in MDA-MB-231 cells transfected with the indicated plasmids were measured by ELISA analysis. (**D**) The protein expression levels of MMP-9 and RABIF as reflected by Western blotting analysis in MDA-MB-231 cells transfected with the indicated plasmids are shown. Actin was served as an internal control. (**E**) Observations of spheres forming under stem cell selective conditions after 8 days of culturing MDA-MB-231 cells transfected with the indicated plasmids. Representative images are shown, and quantitative data are presented as histograms. (**F**) Analysis of cell invasion ability of MDA-MB-231 cells transfected with the indicated plasmids by Boyden chamber assay is shown. The representative photographs of the invaded cells from different treatments are shown as pictures and the quantitative data are shown by histograms. All histograms represent means ± s.e. from three independent experiments (* *p* < 0.05, ** *p* < 0.01). All experiments were performed in triplicates and done at least thrice.

Through enzyme-linked immunosorbent assay (ELISA) analysis, we found that the amount of MMP-2 and MMP-9 decreased upon suppression of RABIF whereas knockdown of FOCAD resulted in increased both MMP-2 and MMP-9 quantities in MDA-MB-231 cells (Figure 6A). Not surprisingly, transfection of FOCAD-exon 4/5 plasmid or overexpression of miR-491-5p induced reduction of both MMP-2 and MMP-9 amount in MDA-MB-231 cells analyzed by ELISA analysis (Figure 6B). As expected, the suppression of both MMP-2 and MMP-9 amount by miR-491-5p can be reverted by cotransfection with miR-491-5p and RABIF in MDA-MB-231 cells by ELISA analysis (Figure 6C). The regulatory function of RABIF and MMP9 was further explored. Knockdown of MMP-9 resulted in attenuation of RABIF protein expression, whereas overexpression of RABIF can partially reverse the inhibition effect by MMP-9 knockdown (Figure 6D). Inhibition of MMP-9 reduced the sphere-forming and cell invasion abilities in MDA-MB-231 cells, and overexpression of RABIF can partly rescue the suppression effect (Figure 6E,F).

From the above data, we can infer that both MMP-2 and MMP-9 are downstream signaling molecules of FOCAD/miR-491-5p/RABIF axis to regulate cancer stemness, drug resistance, cell invasion and metastasis in TNBC.

## 4. Discussion

Treatment options for TNBC are limited due to the lack of therapeutic targets and as a result, are usually managed with chemotherapy such as taxanes and platinum compounds [39]. Treatment of TNBC with taxanes and/or platinum is initially very effective in most patients but rapid recurrence due to chemoresistance is frequently seen and is associated with poor prognosis [6]. Increased amount of evidence suggested that CSCs play a pivotal role in chemoresistance and tumor recurrence [6,33,52], to explore targetable molecule markers regulating cancer stemness and drug resistance is important to improve treatment efficacy in TNBC. In the present study, we unveiled the novel tumor suppressor role of FOCAD/miR-491-5p via targeting RABIF/MMP signaling in TNBC for the first time. We found that miR-491-5p could be an intronic miRNA processed from *FOCAD* gene and first indicated that FOCAD/miR-491-5p inhibit cancer stemness, EMT, drug resistance, cell migration and invasion as well as pulmonary metastasis etc. in TNBC. We are also the first to report that RABIF is the direct target of miR-491-5p to regulate cancer stemness, drug resistance, cell invasion and pulmonary metastasis via MMP signaling in TNBC. Both miR-491-5p and RABIF were found to possess clinical impact on survival in breast cancer and/or TNBC patients. Our study revealed that miR-491-5p and RABIF are influential prognostic biomarkers in breast cancer and TNBC. Targeting the novel FOCAD/miR-491-5p/RABIF/MMP signaling pathway could be a promising strategy to overcome cancer stemness and drug resistance in TNBC to improve treatment efficacy.

MiRNAs have been found to regulate CSCs and the acquisition of EMT phenotype in numerous reports [37,53]. In breast cancer, many miRNAs including miR-200 family, miR-183 family, miR-221, miR-222, miR-142 and miR-214 etc. have been illustrated to target genes and pathways important for maintenance of CSCs and EMT [54,55]. The link among miR-491, CSCs and EMT has also been described in several previous studies. For example, miR-491 was found to attenuate CSCs properties by suppressing GIT-1/NF-κB-mediated EMT in hepatocellular carcinoma (HCC) [56]. Another research also mentioned that miR-491 can target SMAD3 to inhibit CSCs-like properties in HCC [57]. Overexpression of miR-491-5p was depicted to inhibit the propagation of glioma stem cells and to suppress EMT in gastric cancer [58,59]. Nevertheless, the role of miR-491-5p in regulating CSCs and EMT in breast cancer especially in TNBC has never been addressed. Our study first identified that miR-491-5p suppress cancer stemness properties such as sphere forming ability and expression of breast CSC markers in TNBC (Figure 1B,C). Inhibition of EMT shown by various expression levels of EMT markers upon overexpression of miR-491-5p was also first demonstrated in TNBC (Figure 1D). These novel findings depicted that miR-491-5p plays a pivot role in coordinating cancer stemness and EMT in TNBC.

In our experiments, we discovered that miR-491-5p could be an intronic miRNA of *FOCAD* gene (Figure 3A). This finding is in consistent with a previous report to show a deletion at chromosome 9p21.3 which encompasses the *FOCAD* and miR-491 genes in a breast cancer patient [60]. Whether miR-491-5p is actually an intronic miRNA processed from *FOCAD* gene remains unanswered and warrants further investigation. FOCAD protein was first described to be encoded by *KIAA1797* gene as a novel component of the focal adhesion complex to co-localize and interact with vinculin in glioma cells [30]. Deletion of miR-491 as well as its located *KIAA1797* gene was delineated to play a role in the development of early-onset colorectal cancer and breast cancer [60,61]. FOCAD was also found to possess tumor suppressor function to inhibit colony formation, migration, invasion and tumorigenicity in glioma cells [30]. Loss of FOCAD could enhance microtubule assembly and accelerate G2/M phase progression to promote cancer aggressiveness and to worsen clinical outcomes in astrocytic gliomas [62]. Our results which FOCAD functions as a tumor suppressor to inhibit cancer stemness, migration, invasion and pulmonary metastasis are in accordance with the above studies. Both miR-491-5p and FOCAD are first demonstrated to be potential druggable targets in the treatment of TNBC in our study.

Various directly targeted proteins by miR-491-5p in many cancers have been recognized such as hTERT and JMJD2A in cervical cancer [13,14], BCL-XL and EGFR in ovarian cancer [15], Wnt3a and Notch3 in gastric cancer [16,63], TP53 and Bcl-XL in pancreatic cancer [17], G-protein-coupled receptor kinase-interacting protein 1 (GIT1) in OSCC [19] and FOXP4 in osteosarcoma [20]. Although miR-491-5p was also found to target JMJD2B and ZNF-703 to act as a tumor suppressor in breast cancer as mentioned previously [9,10,12], no investigations to pinpoint that RABIF is a direct target of miR-491-5p in breast cancer especially in TNBC in the past. RABIF was once to be identified as a new biomarker candidate of breast cancer development by a bioinformatics tool named PINCAGE (probabilistic integration of cancer genomics data for perturbed gene) [64]. Interestingly, RABIF was found to be structurally similar to translationally controlled tumor protein (TCTP) and TCTP was depicted as prognostic factor and a crucial regulator in CSC compartment in breast cancer [65]. Our data first indicated that RABIF, as a direct target of miR-491-5p (Figure 4B), is a poor prognostic factor in breast cancer and TNBC (Figure 5A,B). We are also the first to elucidate that RABIF functions as an oncogene to promote cancer stemness, drug resistance, cell invasion and pulmonary metastasis which can be reverted by miR-491-5p mediated inhibition in TNBC (Figure 5C–G). We also demonstrated that MMP-2 and MMP-9 are downstream signaling molecules of RABIF (Figure 6A,B). In conclusion, our study shed light on the novel tumor suppressor role of FOCAD/miR-491-5p to target RABIF/MMP signaling in TNBC. This FOCAD/miR-491-5p/RABIF/MMP axis deserves further exploration to develop targeted therapy in the treatment of TNBC.

## Data Availability

Not applicable.

## Supplementary Materials

The following are available online at https://www.mdpi.com/article/10.3390/cells10102524/s1, Figure S1: The mRNA expression levels of miR-491-5p in MCF10A, MDA-MB-231 and Hs578T cells analyzed by qRT-PCR analysis.; Figure S2: qRT-PCR (left) and Western blotting analysis (right) of knockdown efficiency of FOCAD siRNAs in MDA-MB-231 cells.; Figure S3: qRT-PCR analysis of the mRNA expression levels of miR-491-5p upon knockdown of FOCAD in both MDA-MB-231 and Hs578T cells.; Figure S4: The functional role of FOCAD and miR-491-5p.; Figure S5: The functional activity of anti-miR-491-5p on Sphere-forming and cell invasion ability.; Figure S6: Western blotting analysis of knockdown efficiency of RABIF siRNAs in MDA-MB-231 cells.; Figure S7: Western blotting analysis of RABIF protein expression upon transfection with miR-491-5p in MDA-MB-231 and Hs578T cells transfected with pCDNA3.1-RABIF or pCDNA3.1-empty vector.
