# Correction: Huang et al. Identification of the Novel Tumor Suppressor Role of FOCAD/miR-491-5p to Inhibit Cancer Stemness, Drug Resistance and Metastasis via Regulating RABIF/MMP Signaling in Triple Negative Breast Cancer. *Cells* 2021, *10*, 2524

**DOI:** 10.3390/cells13221830

**Published:** 2024-11-06

**Authors:** Wei-Chieh Huang, Hsiang-Cheng Chi, Shiao-Lin Tung, Po-Ming Chen, Ya-Chi Shih, Yi-Ching Huang, Pei-Yi Chu

**Affiliations:** 1 Graduate Institute of Integrated Medicine, China Medical University, NO91, Hsueh-Shih Road, Taichung 40402, Taiwan; jeff20628@gmail.com (W.-C.H.); nonbalance@gmail.com (H.-C.C.); yaoming9@yahoo.com.tw (P.-M.C.); ali5800717@gmail.com (Y.-C.S.); ychuang5176@gmail.com (Y.-C.H.); 2 Chinese Medicine Research Center, China Medical University, NO91, Hsueh-Shih Road, Taichung 40402, Taiwan; 3 Department of Hematology and Oncology, Ton-Yen General Hospital, Hsinchu County 30210, Taiwan; sonoratung@gmail.com; 4 Department of Nursing, Hsin Sheng Junior College of Medical Care and Management, Taoyuan 33858, Taiwan; 5 Graduate Institute of Biomedical Engineering, National Chung Hsing University, Taichung 40402, Taiwan; 6 School of Medicine, College of Medicine, Fu Jen Catholic University, New Taipei 242, Taiwan; 7 Department of Pathology, Show Chwan Memorial Hospital, Changhua 500, Taiwan; 8 Department of Health Food, Chung Chou University of Science and Technology, Changhua 510, Taiwan; 9 National Institute of Cancer Research, National Health Research Institutes, Tainan 704, Taiwan

## Error in Figure

In the original publication [1], there was a mistake in Figure 5 as published. The original Figure 5G has accidentally re-planted the Figure 3E (sh-FOCAD group) image files. The corrected Figure 5 appears below.

The authors stated that the scientific conclusions are unaffected. This correction was approved by the Academic Editor. The original publication has also been updated.

## Figures and Tables

**Figure 5:**
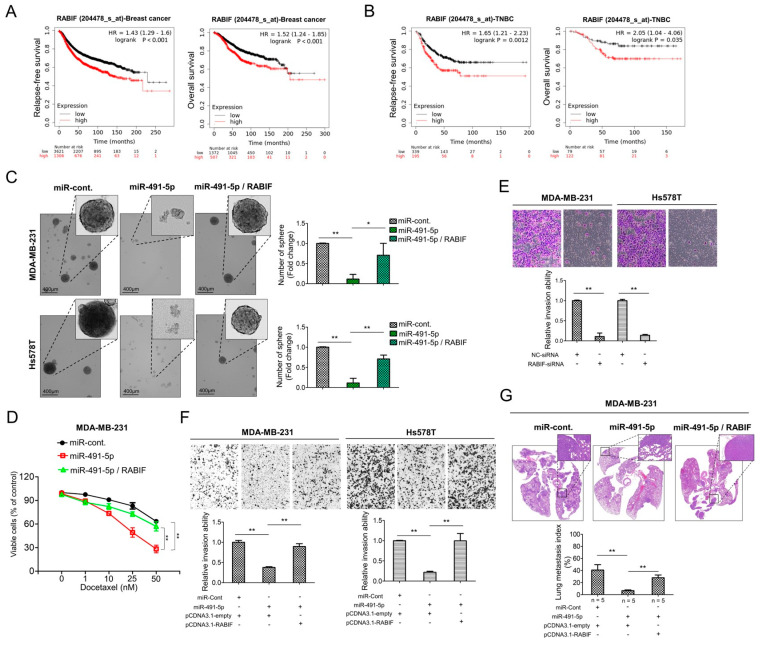
RABIF correlates with poor clinical outcomes and its oncogenic functions can be reverted by miR-491-5p in TNBC. (**A**) Analysis of Kaplan Meier plotter database for RABIF expression in association with relapse-free survival in 4929 breast cancer patients (left) and overall survival in 1879 breast cancer patients (right). (**B**) Analysis of Kaplan Meier plotter database for RABIF expression in association with relapse-free survival in 534 TNBC patients (left) and overall survival in 201 TNBC patients (right). (**C**) Observations of sphere formation under stem cell selective conditions after 8 days of culturing MDA-MB-231 or Hs578T cells transfected with the indicated plasmids (left). Representative images are shown. Quantification of spheres after 8 days of culturing (right). (**D**) MTS assay showing the dose-dependent growth inhibition of MDA-MB-231 cells transfected with the indicated plasmids upon continuous docetaxel exposure at indicated concentrations for 48 h. Each dosage point represents means ± s.e. from three independent experiments (* *p* < 0.05, ** *p* < 0.01). (**E**) Boyden chamber assay showing the effect of RABIF-siRNA on TNBC cells’ invasion ability. Representative photographs of invaded cells from different treatments are shown, and quantitative data are denoted by histograms. (**F**) Boyden chamber assay of the invasion ability of TNBC cells transfected with the indicated plasmids or vectors. Representative photographs show invasive cells under different treatment conditions. Quantitative data are denoted by histograms. (**G**) Lung metastasis of five CB17-SCID mice in each group was monitored after tail vein injection of stably RABIF-overexpressing MDA-MB-231 cells or the control cells which were both transfected with miR-491-5p or the control shown by histologic staining and lung metastasis index. All histograms represent means ± s.e. from three independent experiments (* *p* < 0.05, ** *p* < 0.01).
