# Supplementary material for: Identification of the Novel Tumor Suppressor Role of FOCAD/miR-491-5p to Inhibit Cancer Stemness, Drug Resistance and Metastasis via Regulating RABIF/MMP Signaling in Triple Negative Breast Cancer
Source: Cells. 2021 Sep 24;10(10):2524. doi: 10.3390/cells10102524 (PMC8534268; doi:10.3390/cells10102524)
Supplement: Supplementary file 1 [file cells-10-02524-s001.zip › cells-1334295-supplementary.pdf]

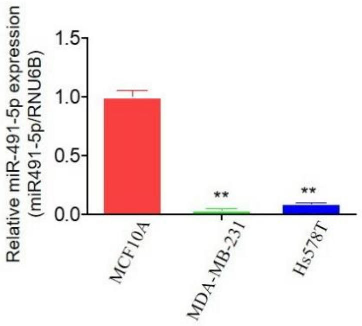

Supplementary Figure S1. The mRNA expression levels of miR-491-5p in MCF10A, MDA-MB-231 and Hs578T cells analyzed by qRT-PCR analysis.

Histograms represent means ± s.e. from three independent experiments (* $p < 0.05$, ** $p < 0.01$). All experiments were performed in triplicates and were done at least three times.

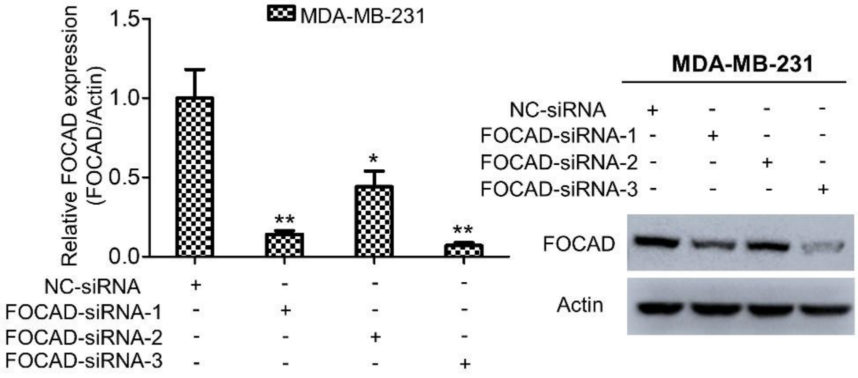

Supplementary Figure S2. qRT-PCR (left) and Western blotting analysis (right) of knockdown efficiency of FOCAD siRNAs in MDA-MB-231 cells.

Left, FOCAD mRNA expressions were normalized to Actin mRNA expressions. Histograms represent means ± s.e. from three independent experiments (* $p < 0.05$, ** $p < 0.01$). Right, Actin was served as an internal control. All experiments were performed in triplicates and were done at least three times.

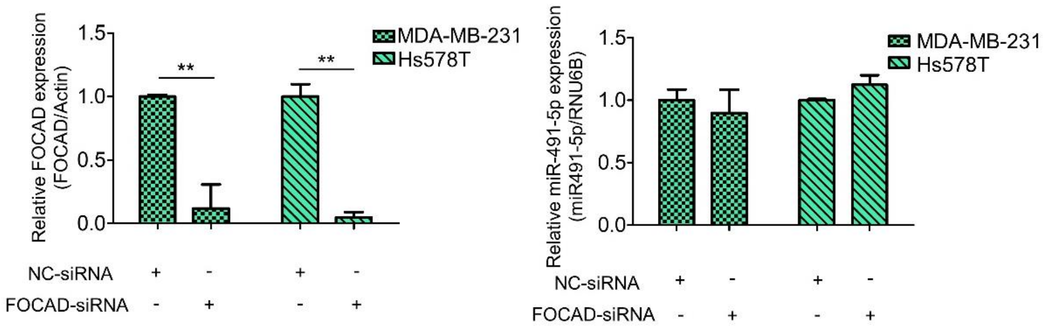

Supplementary Figure S3. qRT-PCR analysis of the mRNA expression levels of miR-491-5p upon knockdown of FOCAD in both MDA-MB-231 and Hs578T cells.

Left, FOCAD mRNA expressions were normalized to Actin mRNA expressions. Right, miR-491-5p mRNA expressions were normalized to RNU6B mRNA expression. Histograms represent means ± s.e. from three independent experiments (* $p < 0.05$, ** $p < 0.01$). All experiments were performed in triplicates and were done at least three times.

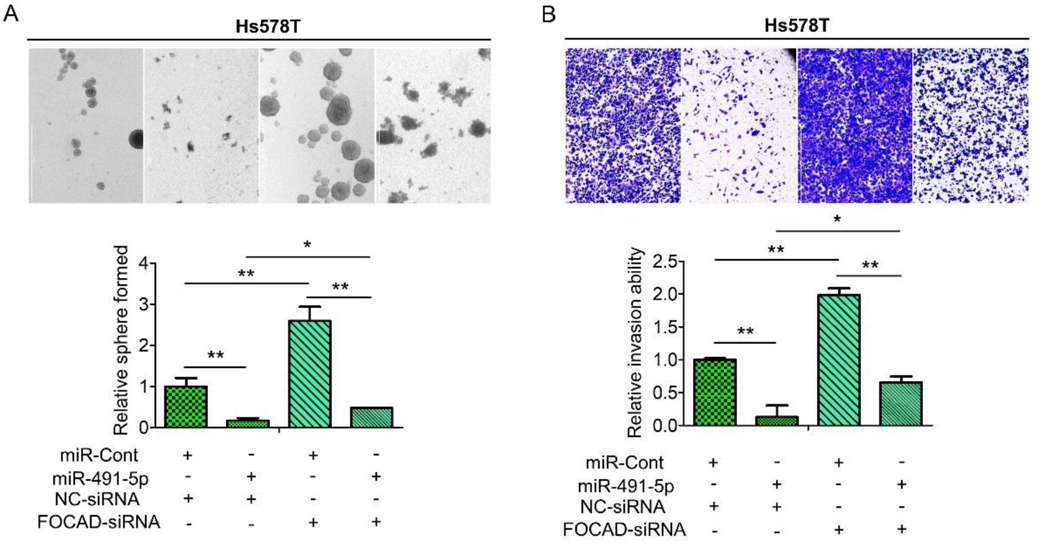

Supplementary Figure S4. The functional role of FOCAD and miR-491-5p.

A. Observations of spheres forming under stem cell selective conditions after 8 days of culturing Hs578T cells transfected with the indicated plasmids. Representative images are shown, and quantitative data are presented as histograms. B. Analysis of cell

invasion ability of Hs578T cells transfected with the indicated plasmids by Boyden chamber assay is shown. Representative photographs of invaded cells from different treatments are shown, and the quantitative data are provided as histograms. Histograms represent means ± s.e. from three independent experiments (* $p < 0.05$, **$p < 0.01$). All experiments were performed in triplicates and were done at least three times.

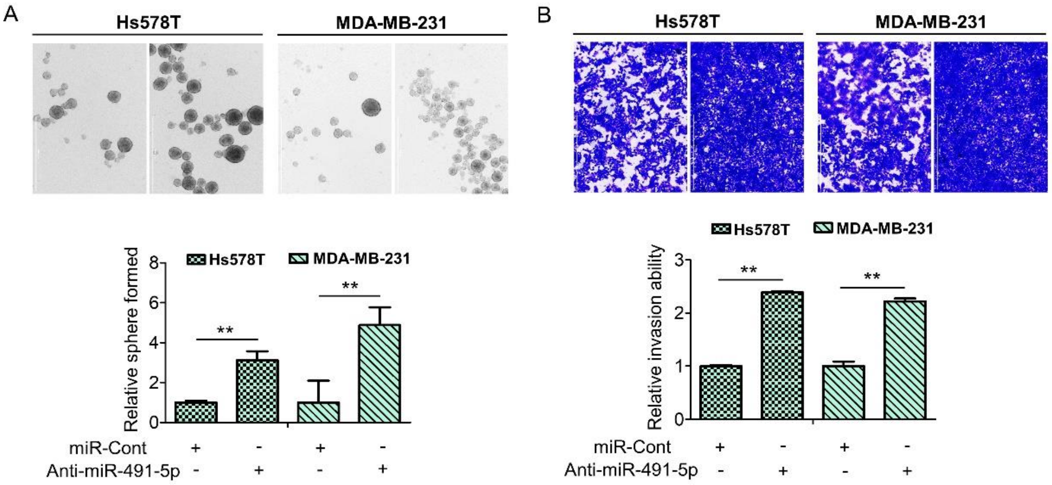

Supplementary Figure S5. The functional activity of anti-miR-491-5p on Sphere-forming and cell invasion ability.

A. Observations of spheres forming under stem cell selective conditions after 8 days of culturing Hs578T and MDA-MB-231 cells transfected with anti-miR-491-5p. Representative images are shown, and quantitative data are presented as histograms. B. Analysis of cell invasion ability of Hs578T and MDA-MB-231 cells transfected with anti-miR-491-5p by Boyden chamber assay is shown. Representative photographs of invaded cells are shown, and the quantitative data are provided as histograms. Histograms represent means ± s.e. from three independent experiments (* $p < 0.05$, **$p < 0.01$). All experiments were performed in triplicates and were done at least three times.

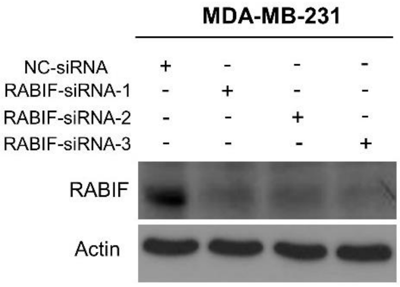

Supplementary Figure S6. Western blotting analysis of knockdown efficiency of RABIF siRNAs in MDA-MB-231 cells.

Actin was served as an internal control. The experiments were performed in triplicates and were done at least three times.

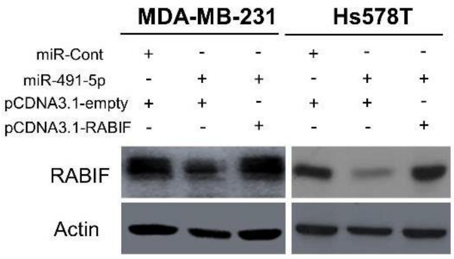

Supplementary Figure S7. Western blotting analysis of RABIF protein expression upon transfection with miR-491-5p in MDA-MB-231 and Hs578T cells transfected with pCDNA3.1-RABIF or pCDNA3.1-empty vector.

Actin was served as an internal control. The experiments were performed in triplicates and were done at least three times.
